# Wind-like sonification of footsteps for walking rehabilitation in chronic stroke survivors: a multiple case study

**DOI:** 10.3389/fpsyg.2026.1874994

**Published:** 2026-10-20

**Authors:** Marta Matamala-Gomez, Jennifer Grau-Sánchez, María F. Porto, Marta A. Rocchetti, Roser Boza, Esther Duarte, Joaquín R. Díaz-Durán, Mohammad Mahdi Dehshibi, Frederic Bevilacqua, Ana Tajadura-Jiménez, Antoni Rodriguez-Fornells

**Affiliations:** 1 Department of Cognition, Development and Educational Psychology, University of Barcelona, Barcelona, Spain; 2 University School of Health and Sport (EUSES), Campus Bellvitge, University of Girona, Girona, Spain; 3 Institute of Neurosciences, University of Barcelona, Barcelona, Spain; 4 Research Group on Complex Health Diagnoses and Interventions from Occupation and Care (OCCARE), Escola Universitària d'Infermeria i Teràpia Ocupacional de Terrassa (EUIT), Universitat Autònoma de Barcelona, Terrassa, Spain; 5 IRCCS San Camillo Hospital, Healthcare Innovation Technology Lab, Venice, Italy; 6 Hospital del Mar Medical Research Institute (IMIM), Barcelona, Spain; 7 Physical Medicine and Rehabilitation Department, Hospital del Mar, Barcelona, Spain; 8 Department of Computer Science and Engineering, Universidad Carlos III de Madrid, Leganés, Madrid, Spain; 9 STMS-IRCAM-CNRS-Sorbonne Université, Paris, France; 10 UCL Interaction Centre, University College London, London, United Kingdgom; 11 Institució Catalana de Recerca i Estudis Avançats, Barcelona, Spain; 12 Aix-Marseille Université, Iméra, Marseille, France

**Keywords:** body perception, chronic stroke, metaphorical sounds, motor rehabilitation, movement sonification, sensorimotor learning, walking recovery

## Abstract

Gait impairments following stroke are prevalent and persistent, often affecting walking ability, body perception, and quality of life. Movement sonification—mapping body motion to sound—has shown promise for modulating sensorimotor function and body movements. This multiple-case experimental study investigated the effects of movement sonification using metaphorical sound on gait, body perception, emotional and cognitive state in three chronic stroke survivors. Using an ABA'C case study design, participants walked with real-time auditory feedback delivered via an interactive walking-interface system (SoniBand) across four periods: baseline (A), “Wind” sound (B or C), return to baseline (A'), and “Mechanical” sound (B or C), presented in a counterbalanced order, each phase lasted 3 weeks. Gait symmetry and walking velocity and ability were assessed as a primary outcome. Further, self-reported body perception, emotional and cognitive state were assessed as a secondary outcome. Exposure to the “Wind” sound condition led to improvements in gait symmetry and walking velocity. Further, showed enhanced perceptions of body lightness, speed, flexibility, mood improvements and sense of pleasure compared to the “Mechanical” sound and baseline periods. The “Mechanical” sound exposure induced a decrease in both gait speed and endurance in one out of the three patients. This multiple-case study provides some preliminary results to support the potential of metaphorical sound sonification to influence movement and body representation for walking rehabilitation in stroke survivors. The results from this case study pave the way to conduct future pilot studies including bigger samples, with a control arm, of chronic stroke survivors presenting walking impairments.

## 1 Introduction

Stroke is a leading cause of acquired disability worldwide, affecting nearly 9.4 million survivors in the United States and over 14.6 million in Europe. Although advances in acute stroke care have improved survival rates, the growing and aging population has led to an increase in stroke incidence and, consequently, a rise in long-term disability and associated economic burden (Wafa et al., 2020; Salbach et al., 2009). Among stroke survivors, 60% to 80% experience impairments in walking, with many losing the ability to walk independently in the acute phase. These deficits often persist, limiting participation in community life and negatively impacting perceived health and quality of life (Salbach et al., 2009). Walking impairments after a stroke are typically characterized by reduced gait speed, cadence, and stride length, as well as asymmetry between the lower extremities in spatiotemporal parameters (Brouwer et al., 2009). In addition, stroke survivors frequently experience somatosensory deficits and sensory loss, contributing to altered body perception (van Stralen et al., 2011), as explained before. Indeed, a recent study reported internal changes in stroke survivors such as diminished sensitivity, decreased proprioception, tingling sensations, and fatigue (Vinje and Normann, 2025). Moreover, stroke survivors described the challenges of not being able to move certain body parts as they did as heathy. Especially, regarding walking movement, they referred body parts as a ‘semi-lifeless limb', and ‘the rigid foot that needs to be swung forward while walking', leading to negative emotional responses (Vinje and Normann, 2025).

In relation to walking ability, it is supported by an extensive neural network involving the spinal cord, brainstem, cerebellum, basal ganglia, limbic system, and cerebral cortex. These structures interact dynamically with environmental inputs (Takakusaki, 2013). Particularly, the cerebellum plays a key role by integrating feed-forward signals and real-time sensory feedback from multiple sensory modalities (visual, auditory, vestibular, tactile, and proprioceptive) to support adaptive locomotion (Morton and Bastian, 2004). After a stroke injury, structural and connectivity changes in the sensorimotor cortex are common. Animal studies have shown an increase in somatosensory-evoked potential in the contralesional cortex within the first hours after brain injury (Dancause et al., 2015). This somatosensory dysfunction can result in altered internal body representations of the affected side (Berlucchi and Aglioti, 2010), which, in turn, may lead to impaired proprioceptive and kinesthetic processing and distortions in the space around the body (Wallwork et al., 2016). Since motor control relies on continuous sensorimotor loops and predictive models of movement outcomes, disruptions in somatosensory processing affect motor planning, execution, and adaption (Wolpert and Ghahramani, 2000). Hence, mismatches between predicted and actual sensory feedback, known as disruptions in internal forward models, impair motor function (Roerdink et al., 2009). Indeed, stroke survivors often exhibit reduced postural steadiness and asymmetry of weight, leading to postural imbalance. In particular, stroke survivors frequently report an increased perception of weight on the non-paretic leg, especially in those with ankle clonus, disturbed sensibility, and impaired selective muscle control on the paretic side (Bevilacqua et al., 2016). Such weight imbalance between the lower extremities leads to reduced postural steadiness and asymmetry of weight bearing, which in turn may provoke changes in body perception (Bevilacqua et al., 2016). To address all the sensorimotor and subsequent body alterations after suffering a stroke injury, new sensory-based learning rehabilitation techniques represent an avenue to update internal forward models by manipulating sensory inputs, signaling the motor system to adjust and minimize discrepancies in predicted and actual feedback.

One strategy is movement sonification, which maps body movements onto sound in real time. This approach provides auditory feedback about the body position to support proprioception, increase body awareness, guide movement execution, deliver performance feedback, and facilitate sensorimotor learning (Brodie et al., 2015). In neurological rehabilitation, real-time auditory feedback has been used to support gait training by cueing steps and compensating for proprioceptive deficits (Pitale and Bolte, 2018; Baram and Miller, 2007; Tajadura-Jiménez et al., 2015). Auditory feedback can also serve as a means of sensory alteration or distortion of one's own body perception, which in turn can alter motor performance and emotional state. For example, the study by Tajadura-Jiménez et al (Gomez-Andres et al., 2020) demonstrated that altering the natural auditory feedback of one's own footsteps, specifically by modulating the sound frequency spectra, can induce the illusion of having a lighter or heavier body, a phenomenon known as the “Footsteps Illusion.” These shifts in body perception are accompanied by measurable changes in emotion and gait, as recorded by sensors and self-reports (Gomez-Andres et al., 2020). In chronic stroke survivors, modifying the auditory feedback of one's own footsteps via frequency shifts has been found to influence gait symmetry, with low-frequency augmentation improving stride and stance time symmetry, and high-frequency augmentation producing the opposite effect (Ley-Flores et al., 2021a). Beyond modifying the actual sounds produced by the body, other studies targeting alterations of body perception have explored the use of metaphorical or figurative sounds (i.e., naturalistic or environmental sounds) such as wind, water, or mechanical gear sounds, synchronized with movement. These studies aim to link the metaphorical qualities of such sounds to perceptions of the body and movement (e.g., wind may be associated with flexibility or faster movements), and in turn influence actual movement behavior and engagement in physical activity (Ley-Flores et al., 2022; Turmo Vidal et al., 2024; Ley-Flores et al., 2019). Research using these types of sounds have shown that they can lead to changes in perceived physical capabilities (e.g., feeling stronger, more flexible, or agile), movement quality (e.g., acceleration) and physical exertion (e.g., the number of repetitions in strengthening or flexibility exercises) among people who are physically inactive (Ley-Flores et al., 2022; Grau-Sánchez et al., 2017). For instance, wind sounds provided synchronously to body movements during physical activity have been found to enhance the sense of agency, improve perceived movement flexibility, and increase the feeling of physical capability compared to other sound stimuli such as mechanical gear sounds or generic beeps (Ley-Flores et al., 2022). Despite these promising findings related to physical activity and movement perception, the use of metaphorical sounds for movement sonification has yet to be evaluated in clinical populations with gait impairments, such as stroke survivors.

To this aim, the present single-case experimental study aimed to examine the potential effect of sonifying gait with different metaphorical sounds to improve body perception and walking ability in three chronic stroke survivors presenting lower limb hemiparesis. Specifically, we examined the effects of two types of metaphorical sounds (“Wind” and “Mechanical”), compared to self-produced natural footstep sounds, delivered through an interactive walking interface system [SoniBand; (Ley-Flores et al., 2022)]. The primary endpoint is to assess the impact of the selected metaphorical sounds (“Wind” and “Mechanical”) on gait symmetry and walking velocity. The secondary endpoints are to assess changes in body and walking movement perception, and cognitive performance following exposure to the sound stimuli. We hypothesized that footstep sonification would improve walking ability compared to self-produced natural footstep sounds in chronic stroke survivors. We also hypothesize that different sounds (“Wind” and “Mechanical”) would yield distinct effects on the primary and secondary endpoints. Specifically, based on prior work with physically inactive populations (Ley-Flores et al., 2022; Krasny-Pacini and Evans, 2018), we expected the “Wind” sound to produce the greatest effects in walking ability and body perception compared to the “Mechanical” and to the self-produced natural footstep sounds).

## 2 Materials and methods

### 2.1 Study design

This study employed a single-case experimental design with three chronic stroke survivors (Kwong and Ng, 2019; Dalrymple-Alford et al., 2010). The design consisted of 4 intervention periods alternating control and experimental conditions (ABA'C, Figure 1) to compare the effects of different movement sonification stimuli delivered by the SoniBand system.

**Figure 1:**
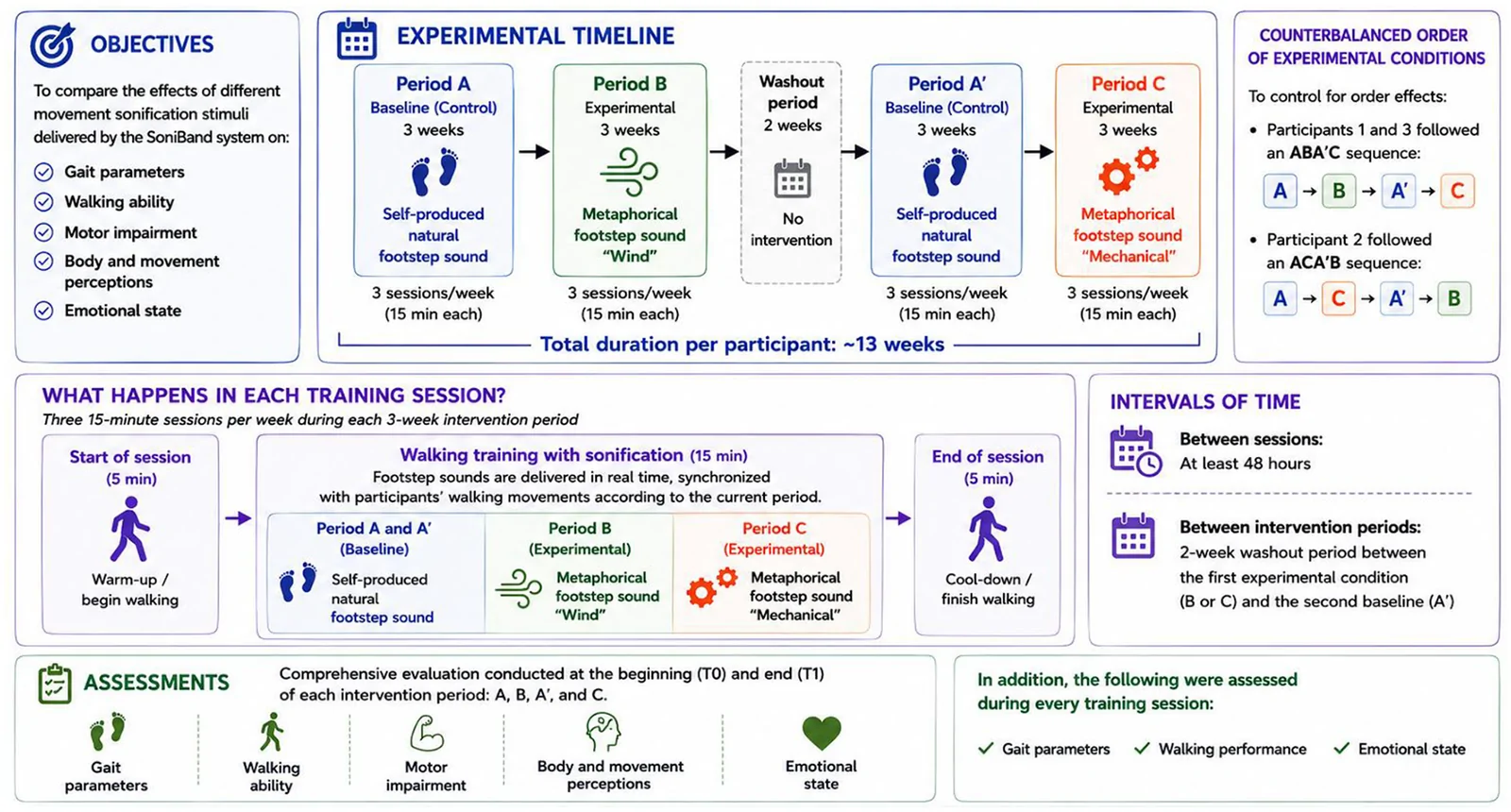
Experimental study design. Representation of the study design (ABA'C), objetives and assessment measures, in which different sound stimuli were presented to the participants synchronously to their footstep movement while walking along a defined path in periods B and C. The sound stimuli, “Wind” or “Mechanical” sounds were counterbalanced among the three chronic stroke survivors'. In periods A and A', there was no sound stimuli provided while walking in the same defined path. During the no sound periods (A and A') participants heard their own self-produced footstep sounds. All three participants conducted the three interventions ABA'C.

Each intervention period lasted 3 weeks, during which participants engaged in a structured walking training program consisting of three 15-min sessions per week. The *A* periods served as baseline-control conditions, during which participants received self-produced natural footstep sound. In the *B* and *C* periods, participants received metaphorical footsteps sounds (“Wind” or “Mechanical”), synchronized with their walking movements. The order of *B* and *C* conditions was counterbalanced across participants for control order effects: participants one and three followed an ABA'C sequence, while the second one followed an ACA'B sequence. To minimize carryover effects, a 2 week washout period was introduced between the first experimental condition and the second *period A'*.

Baseline assessments were carried out to describe participants. In addition, a comprehensive evaluation was conducted at the beginning (T0) and end (T1) of each period to assess gait parameters, walking ability, motor impairment, perceptions of body and movement, and emotional state. In addition, gait parameters, walking performance, and emotional state were assessed during every training session (Table 1).

**Table 1:** Assessment measures and assessment periods.

Outcome measures	T0 first day of each period	T1 last day of each period	t1, t2, t3, t4, t5, t6 3 weeks
Gait performance			
10-Meter Walk Test.	**•**	**•**	
6-MinWalk Test	**•**	**•**	
Walking parameters from Soniband (Walking Symmetry, Step Frequency, and Acceleration Intensity)	**•**	**•**	**•**
Executive function while walking			
Walking Trail-Making Test (WTMT)	**•**	**•**	
Perception and sensory integration			
Sound perception questionnaire	**•**	**•**	**•**
Body and movement feelings **(only sound periods B and C)**	**•**	**•**	
Emotional and affective state			
Self-Assessment Manikin Scale.	**•**	**•**	**•**
Profile of Mood States (POMS)	**•**	**•**	

### 2.2 Participants

Three chronic stroke survivors presenting lower-limb hemiparesis and impaired walking ability were included in the study. In order to be eligible for inclusion, the following criteria was considered: (i) slight-to-moderate paresis of the lower extremity after a first-ever stroke, defined as having a score from < 21 to 34 in the Fugl-Meyer Assessment of Motor Recovery (Senda et al., 2020) (indicating lower mobility function); (ii) no major cognitive deficits affecting language comprehension, such as difficulties in understanding task instructions or questionnaire statements, as indicated by a Montreal Cognitive Evaluation score ≥26 (Jiskoot et al., 2023); and (iii) absence of other psychiatric or neurological comorbidities; (iv) absence of auditory impairment. Participants were recruited from a database of individuals who had previously undergone outpatient rehabilitation at the Department of Rehabilitation and Physical Medicine at Hospitals del Mar i l'Esperança (Barcelona, Spain). The study was approved by the ethics committee Bellvitge University Hospital (reference num. PR221/23; L'Hospitalet del Llobregat, Spain) and Hospital del Mar (Barcelona, Spain). All participants provided written informed consent prior to enrollment.

### 2.3 Equipment: movement sonification system

SoniBand is a wearable device developed to sonify lower-limb movements in real time, used in previous studies on body perception (Grau-Sánchez et al., 2017). The system consists of a wearable band made of hand-sewn cloth pocket housing a wireless motion sensor (BITalino R-IoT), embedding a 9-axis Inertial Motion Unit (IMU) digitized at 16 bits and sampled at 100 Hz. The band transmits motion data using Wi-Fi to a program created in the Max8 (Cycling'74) environment using the MuBu package (IRCAM). The program enables the processing of the motion data, and a user interface allows for selecting and customizing movement sonification parameters. In our case, we chose to generate real-time auditory feedback by triggering “Wind” and “Mechanical” sounds on the accelerometer onsets (above an adjustable threshold). Participants wear wireless headphones (Sennheiser RS 120-W) to receive auditory feedback, thus synchronized with their footstep movements, enabling an immersive and ecologically valid auditory experience (Figure 2). Simultaneously, the system records and stores movement data for further analysis.

**Figure 2:**
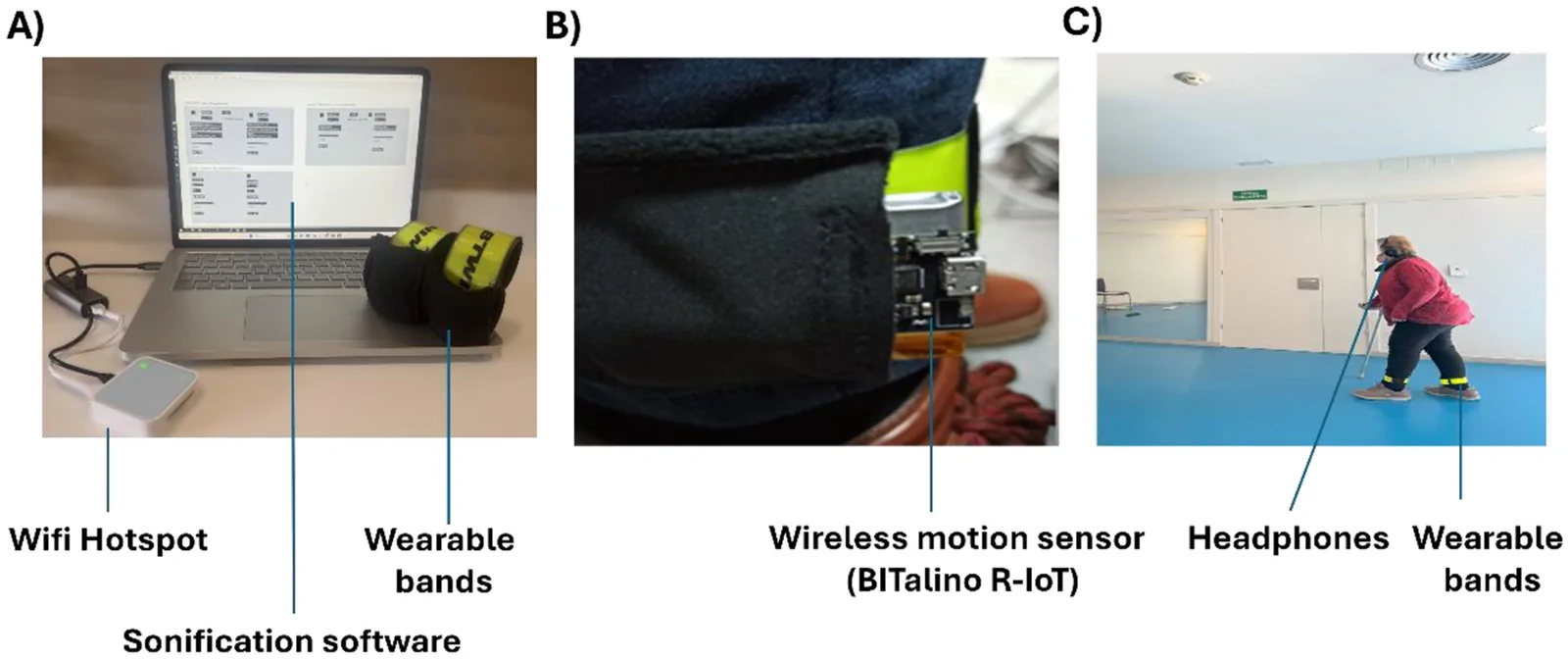
SoniBand system and individual with chronic stroke walking wearing the system. **(A)** The picture shows the wearable band made of hand-sewn cloth pocket housing a wireless motion sensor (BITalino R-IoT), the Wifi Hotspot device, and the software platform created in the Max8 (Cycling'74) environment using the MuBu package (IRCAM). **(B)** The picture shows the BITalino R-IoT wireless motion system. **(C)** The picture shows chronic stroke survivors performing the training session at the hospital.

### 2.4 Sound conditions

Two different sonification conditions were chosen with different meaning and sound characteristics: “Wind” and “Mechanical,” based on previous results (more detail about the sound input files here: https://osf.io/wh8dr/files/osfstorage). In particular, “Wind” aims to build on feelings of movement flexibility, effortless speed and agency, based on (Ley-Flores et al., 2019, 2021a,b); “Mechanical” aims to enhance control/proprioception, based on (Singh et al., 2016; Ley-Flores et al., 2021a).

The “Wind” sonification plays a pink noise sound which increases or decreases its frequency (starting at a frequency of 220 Hz) according to the acceleration of the foot during the swing phase of a step. The “Mechanical” sonification plays discrete sounds, mimicking the sound of rusty gears; it plays through the movement with gradual changes in frequency and speed according to the acceleration of the foot. Hence, it should be noted that beyond the different metaphorical associations triggered by the nature of the sound, there are different movement related information embedded in the sound.

### 2.5 Sonification procedures

Each footstep was detected by the SoniBand system, which triggered the corresponding auditory stimulus in temporal synchrony with the participant's walking movement. The primary movement-related parameter controlling sound delivery was a step onset. The acoustic characteristics of the sounds were predefined according to the experimental condition: self-produced natural footstep sounds during the baseline conditions and metaphorical “Wind” or “Mechanical” footstep sounds during the experimental conditions. No additional gait parameters were used to continuously modulate the acoustic characteristics of the sounds.

### 2.6 Training procedures

During all the training sessions, the participants were using their usual walking devices. At the beginning of each session, the SoniBand system was attached to both ankles of participants to record gait parameters in real time. In the sessions with sound, they wore wireless headphones to deliver the auditory stimuli in an immersive manner while isolating external sounds. In the sessions with sound, with each step, the system generated a trigger that activated the sound output via the dedicated software interface (see section 2.3 for further equipment details). In all sessions, walking took place along a defined path within a 64 m^2^ quiet room, minimizing external distractions. To reduce the risk of fatigue, each 15-min session was divided into three walking bouts, separated by two rest periods. During rest, participants were instructed to remain seated and avoid moving their legs or feet.

Participants began with the A period (baseline), which lasted 3 weeks and consisted of three walking training sessions per week, each approximately 15 min long. During this period, participants walked at a self-selected pace while listening to the natural sound of their own footsteps (self-produced natural footstep sound). In the A period serving as a baseline, although participants wore the SoniBand sensors, no metaphorical sound was reproduced; only the natural footstep sound was played, and gait parameters were recorded. Following the A period, participants engaged in experimental periods (B and C), during which metaphorical sounds were delivered synchronously with their footstep movements. In the B period, the “Wind” sound was presented, to generate the illusion of walking in the wind. In the C period, the “Mechanical” gear sound was delivered, to generate the illusion of mechanical movement.

### 2.7 Evaluation of participants

As movement sonification is a multidimensional technique which can have an impact on different dimensions of the participants such as quality of the movement, movement performance, and emotional state, baseline and outcome assessment included multidimensional assessment measures. Participant evaluation included a multidimensional baseline assessment to comprehensively characterize their profiles at the start of the study. Outcome measures were administered at different time points, with some collected at the beginning and end of each period and others following each training session (see Table 1). These measures were used to assess gait performance, executive function during walking, perception, and emotional state.

#### 2.7.1 Baseline assessments

Baseline assessments were conducted to evaluate participants' cognitive functioning, functional status, and emotional wellbeing, with the aim of characterizing the sample prior to the intervention. To evaluate visuospatial abilities and attentional capacity, we administered Addenbrooke's Cognitive Examination (ACE) (Grégoire and Van der Linden, 1997), the Benson Complex Figure Test (immediate and delayed visual memory) (Perrochon and Kemoun, 2014), the Digit Span Forward and Backward tasks (Shelkey and Wallace, 2000), and the Walking Trail Making Test (WTMT) (Mahoney, 1965), the WTMT was also used as an outcome measure (see section 2.5.2).

Activities of daily living (ADL) and functional mobility were assessed using the Katz Index of Independence in Activities of Daily Living (Wei and Hodgson, 2023), the Barthel Index (Rosario et al., 2022), the Lawton and Brody Instrumental Activities of Daily Living Scale (Bertoni et al., 2018), and the Fall Risk Assessment Scale (Duncan et al., 2014). In addition, we evaluated walking ability using the Modified Walking Ability Categories (Brañez-Condorena et al., 2021) and lower-limb motor function with the Fugl-Meyer Assessment (lower limb section) (Sun et al., 2021).

For the evaluation of emotional well-being, depressive symptoms were assessed using the Geriatric Depression Scale (GDS) (Monteiro et al., 2022), anxiety levels were evaluated with the Generalized Anxiety Disorder Scale (GAD-7) (Gafner and Bruyneel, 2022), and global self-esteem was measured using the Rosenberg Self-Esteem Scale (Kubo et al., 2020).

#### 2.7.2 Outcome measures

There will be pre-post assessments times for each stage, as well as daily assessment during all ABA'C periods (Figure 2). In specific, as a primary outcome we evaluated walking parameters extracted from the RIOTs (Walking Symmetry, Step Frequency, and Acceleration Intensity) as well as the walking ability assessed by functional scales. As a secondary outcome, we also assessed possible changes in body perception (specifically in the paretic side), the level of pleasure after performing the training sessions, the sound perception, and other cognitive and emotional factors (see Table 1).

##### 2.7.2.1 Gait performance

The 10-Meter Walk Test (10-MWT), (Horsak et al., 2016) (Gafner and Bruyneel, 2022) and the 6-Minute Walk Test (6-MWT), (Bradley and Lang, 1983) were administered at the beginning and end of each intervention period (T0 and T1), without sonification. The first test measures walking speed by over 10 meters (meters/seconds) at a comfortable pace, while the second assesses walking endurance by recording the distance walked in 6 min (meters). Walking parameters from the SoniBand system were collected during each sonification walking session (t1 – t6). Data included gait symmetry, step frequency, and acceleration intensity, recorded through the Soniband system and its integrated accelerometer/gyroscope sensors.

##### 2.7.2.2 Executive function while walking

We used the Walking Trail-Making Test (WTMT), an adapted version of the traditional Trail Making Test (TMT) for walking conditions (Mahoney, 1965) at baseline (period A, T0) and after the sound interventions (period B and C, T1; see Figure 3). The WTMT includes three conditions, (i) assessing attentional control during walking by requiring participants to step on numbered targets (1–20) in ascending order, without a secondary cognitive task (W-TMT-N); (ii) evaluating selective attention while walking, with participants stepping on ascending numbers (1–20) while ignoring distractor targets (W-TMT-A); and (iii) a dual-task condition assessing executive function (working memory and cognitive flexibility) by requiring participants to alternate between numbers and letters in sequence (1–A−2–B…) while avoiding distractors (W-TMT-B).

**Figure 3:**
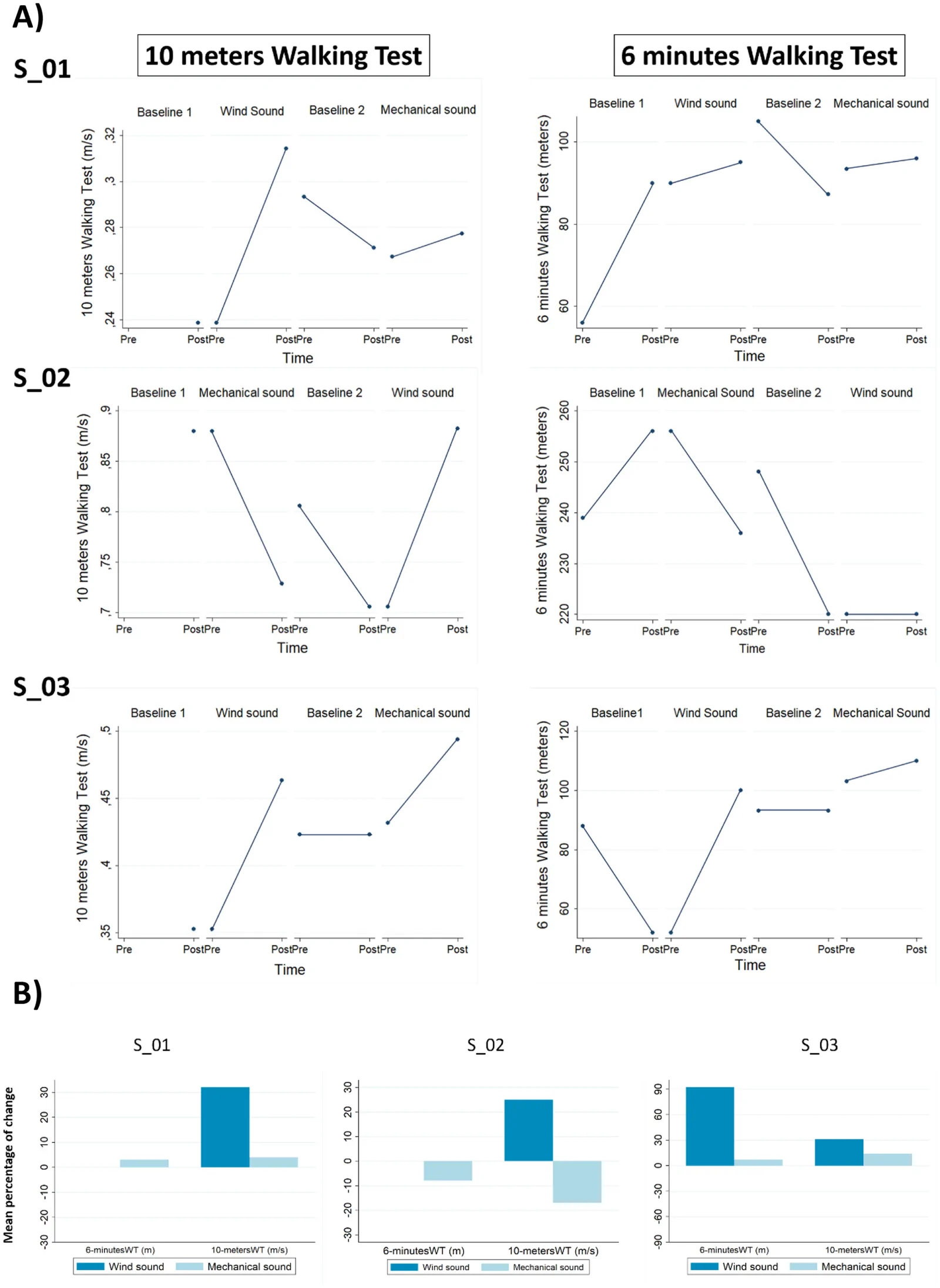
Results of clinical walking tests. **(A)** Results from the 10 meters walking test by subjects from baseline (T0, pre) to the post-training time (T1) in each training period (left side). Results from the 6-min walking test by subjects from baseline (T0, pre) to post-training time (T1) in each training period (right side). **(B)** Percentage of change from Baseline 1 and 2 to post-training time in “Wind” and “Mechanical” sound trainings by subject in the 6-min walking test and 10-meters walking test. Overall, the results show an improvement in walking ability in terms of velocity (m/s) in the 10-meters walking test, and in walking distance with the 6-minutes walking test, with the “Wind” sound condition in all three subjects. In periods of Baseline 1 **(A)** and Baseline 2 (A'), the chronic stroke survivors were hearing their own footsteps.

##### 2.7.2.3 Sound Perception and Body and movement feelings

Sound perception was assessed using the sound perception questionnaire adapted from (Shacham, 1983), evaluating their subjective response to the auditory stimuli. The first, rated the level of agreement on a 4-point Likert scale (1 = not agree to 4 = fully agree), measured seven aspects of sound perception: (1) awareness of one's walking pattern, (2) synchrony between sound and footstep movement, (3) synchrony with ankle movement, (4) perception of walking rhythm, and the perceived impact of sound on (5) walking speed, (6) walking rhythm, and (7) ankle movement (see Supplementary Material for details).

At the end of each sound intervention period (B and C), participants also completed a second questionnaire adapted from (Turmo Vidal et al., 2024), assessing the level of agreement toward different body and movement feelings using a 7-point Likert scale. Six items addressed body-related feelings during walking: (1) Weight: “*Light*” to “*Heavy*”; (2) Strength: “*Weak”* to “*Strong”*; (3) Capability: “*Not able”* to “*Able”*; (4) Flexibility: “*Not flexible*” to “*Flexible*”; (5) Speed: “*Slow”* to “*Fast”*; (6) Tiredness: “*Not tired”* to “*Tired”;* and two items assessed perceived control and movement pace: (1) Control: “*Not in control*” to “*In control*”; and (2) Acceleration: “*Accelerated*” to “*Decelerated”*. Full item descriptions are provided in the Supplementary material.

##### 2.7.2.4 Emotional and affective state

After each walking session (t1 – t6), participants rated their experience using the Self-Assessment Manikin Scale (Berry and Ayers, 2006), which measures emotional response in terms of pleasure, arousal, and dominance. In addition, mood changes across the intervention were assessed using the Profile of Mood States (POMS) (Ugille et al., 2012), a multidimensional tool that captures six transient mood states: tension–anxiety, depression–dejection, anger–hostility, vigor–activity, fatigue–inertia, and confusion–bewilderment.

### 2.8 Analyses

For the clinical standardized motor test (10-mWT and 6-MWT), we obtained 8 data points (4 periods). As the results did not show any minimal clinical detectable difference, we calculated the percentage of change from the baseline (T0, period A) to the post assessment time (T1) for both conditions “Wind” and “Mechanical” sound. The formula to calculate the percentage of change is the following (Melzer et al., 2009): % of change = (Post training (T1)-Baseline (T0))/ Baseline x 100.

For the tests in which we collected repeated measures (daily measures) we employed a mixed-effects design (Peña-Casanova et al., 2009) analyze our dependent variables, considering four levels as fixed effects: sound condition (no sound (baseline 1), “Wind” sound, no sound (baseline 2) “Mechanical” sound) and random effects over the individual subjects. In specific, we analyzed differences in the walking parameters extracted from the RIOTs (Walking Symmetry, Step Frequency, and Acceleration Intensity), using the multilevel mixed effect linear regression (*mixed* function for continuous quantitative measurement data in Stata) by ID. We used the following command in Stata: *by ID, sort: mixed Outcome variable i.Condition* (four levels: baseline1, wind sound, baseline 2, mechanical sound), providing three independent regressions by ID. The sound perception questionnaire and the Self-Assessment Manikin scale across conditions were assessed uisng the multilevel mixed effect ordered logistic regression test (using the *meologit* function in Stata). The significance level was set at *p* < 0.05. Furthermore, a pairwise comparison between sound conditions was performed using model-based Wald contrasts implemented with Stata's pwcompare post-estimation command. Pairwise contrasts were reported as z statistics and two-sided p-values. We used descriptive analyses for the pre-post (T0-T1) measures: the *10-Meter Walk* Test, the *6-Minute Walk Test*, the *Walking Trail-Making Test (WTMT)*, and the *Body and Movement feelings questionnaire*.

To explore the impact of auditory cues on walking asymmetry in stroke survivors, we assessed gait symmetry by comparing step frequency and acceleration intensity between the affected and non-affected lower limbs. Gait metrics, including step frequency, step intensity, and symmetry indices, were derived from accelerometer data using a systematic signal processing approach.

To evaluate gait characteristics in stroke survivors, we developed a methodology to analyze walking patterns using wearable accelerometer sensors placed on both lower limbs (Sepas-Moghaddam et al., 2012). The sensors, sampled at a frequency of 100 Hz, captured acceleration intensity data during walking tasks. This acceleration of intensity reflects the dynamic movements of each foot. Our signal processing pipeline applies a low-pass filter at [100 Hz] prior to extracting the acceleration intensity and symmetry variables, which attenuates the high-frequency heel-strike transients and any potential clonic/tremor-band artifacts (typically 3–12 Hz) while preserving the gait-cycle-relevant acceleration envelope used for our metrics. We have added this filtering detail to the Methods, as it was previously underspecified. The proposed method quantifies key gait parameters, which are crucial for assessing motor recovery and symmetry in stroke rehabilitation. These gait parameters include step frequency and step intensity, which correspond to the number of steps per second and the magnitude of acceleration peaks during steps, respectively. We developed a peak-detection algorithm specifically tailored to the gait patterns of stroke survivors to identify acceleration peaks, i.e., individual steps. To ensure the accuracy of step identification, we applied a minimum peak distance of setting it to 1.295 msec (Matías-Guiu et al., 2018), to filter out noise and capture true steps. Peaks were further refined by focusing on the frequency range of 0.4-1.8 Hz, which is characteristic of walking in stroke survivors, and by setting a dynamic threshold based on the signal's power spectrum to distinguish significant steps from background noise. This process was applied separately to data from the left and right limbs, allowing for the identification of steps for both the affected and unaffected sides. Further, if some peaks were missed, we used the “*findpeaks”* function in Matlab to ensure all the peaks were selected and counted for the analyses.

To quantify gait symmetry, we calculated two primary metrics: step frequency and step intensity symmetry. Step frequency was determined by dividing the number of detected steps by the total active walking time, defined as periods with steps occurring less than 1 min apart. Step intensity was computed as the average magnitude of acceleration peaks, reflecting the forcefulness of each step. Symmetry indices were derived by comparing these metrics between the affected and non-affected limbs, using the ratio of the minimum to the maximum value for both step frequency and intensity. A ratio closer to 1 indicates greater symmetry, while lower values suggest asymmetry, which is common in stroke survivors due to motor impairments. Active walking periods were distinguished from rest periods (gaps exceeding 1 min between steps) to focus the analysis on functional movement, providing a clearer picture of gait performance during rehabilitation.

## 3 Results

### 3.1 Baseline assessments results

Tables 2, 3 describe participants' basic demographic and clinical variables, along with baseline evaluations of cognitive functioning, functional status, and emotional wellbeing. The three participants differed in age, time since stroke onset, and side of body affected, allowing for the observation of individual variability in response to the intervention. Baseline assessments revealed diverse cognitive and functional profiles, with notable differences in executive function, sustained attention, and independence in daily living. Due to the small sample size (*N* = 3) the results should be framed as observations rather than evidence of efficacy.

**Table 2:** Demographic and Clinical characteristics of participants.

Subjects	Gender	Age	Time since the onset stroke	Affected hemisphere	Affected side of the body
S_01	Male	84	1 year	Right	Left
S_02	Female	75	2 years	Left	Right
S_03	Male	60	4 years	Right	Left

**Table 3:** Results from baseline evaluation on cognitive functioning, functional status, and emotional wellbeing.

Domain Cognitive functioning	Test [Percentile o range Pe]	S_01 [Pe]	S_02 [Pe]	S_03 [Pe]
Brief Cognitive Screening	Montreal Cognitive Assessment	26 (Dalrymple-Alford et al., 2010)	29 (Dalrymple-Alford et al., 2010)	27 (Dalrymple-Alford et al., 2010)
Global cognitive screening	ACE [≥41-59^**b**^]	74 (Grégoire and Van der Linden, 1997; Perrochon and Kemoun, 2014; Shelkey and Wallace, 2000; Mahoney, 1965; Wei and Hodgson, 2023; Rosario et al., 2022; Bertoni et al., 2018; Duncan et al., 2014; Brañez-Condorena et al., 2021; Sun et al., 2021; Monteiro et al., 2022; Gafner and Bruyneel, 2022)	89 (Lesur et al., 2025; Stanton and Spence, 2020; Tajadura-Jiménez et al., 2022; Navas-León et al., 2023; Rizzo et al., 2015; Scholz et al., 2015; Potapov et al., 2025; Scholz et al., 2016; Turmo Vidal et al., 2025; Petreca et al., 2023; Ley-Flores et al., 2021a; Botvinick and Cohen, 1998)	81 (Takakusaki, 2013; Morton and Bastian, 2004; Dancause et al., 2015; Berlucchi and Aglioti, 2010; Wallwork et al., 2016)
Immediate visuospatial memory	Benson Copy [≥40^c^]	13 [>5]	15 (Ley-Flores et al., 2022)	14 (Vinje and Normann, 2025)
Delayed visuospatial memory	Benson delay [≥40^c^]	12 (Perrochon and Kemoun, 2014)	10 (Wallwork et al., 2016; Wolpert and Ghahramani, 2000)	15 (Ley-Flores et al., 2021a)
Control attention: Sustained attention	Digit span forward [≥41-59^**a**^]	3 (Salbach et al., 2009)	7 [98]	5 (Kubo et al., 2020; Horsak et al., 2016; Bradley and Lang, 1983; Shacham, 1983; Berry and Ayers, 2006; Ugille et al., 2012; Melzer et al., 2009; Peña-Casanova et al., 2009; Matías-Guiu et al., 2018; Bernat et al., 2015; Jiménez-Caballero et al., 2012; De et al., 2002; Kapoor et al., 2019; Park and Kim, 2016; Tajadura-Jiménez et al., 2012, 2019; Tajadura Jimenez et al., 2015; D'Adamo et al., 2024; Tajadura-Jiménez et al., 2017)
Woking memory and inhibitory control	Digit span backward [≥41-59^**a**^]	2 (Ley-Flores et al., 2021a, 2022; Turmo Vidal et al., 2024; Ley-Flores et al., 2019; Grau-Sánchez et al., 2017; Krasny-Pacini and Evans, 2018; Kwong and Ng, 2019; Dalrymple-Alford et al., 2010; Senda et al., 2020; Jiskoot et al., 2023)	5 (Spence, 2011, 2019; Keus et al., 2012; Roel Lesur et al., 2025; Lesur et al., 2025; Schmitz et al., 2018)	4 (Lesur et al., 2025; Stanton and Spence, 2020; Tajadura-Jiménez et al., 2022; Navas-León et al., 2023; Rizzo et al., 2015; Scholz et al., 2015; Potapov et al., 2025; Scholz et al., 2016; Turmo Vidal et al., 2025; Petreca et al., 2023; Ley-Flores et al., 2021a; Botvinick and Cohen, 1998)
Control attention: Sustained attention and processing speed	TMT part A [≥41-59^**a**^]	84 (Ley-Flores et al., 2021a, 2022; Turmo Vidal et al., 2024; Ley-Flores et al., 2019; Grau-Sánchez et al., 2017; Krasny-Pacini and Evans, 2018; Kwong and Ng, 2019; Dalrymple-Alford et al., 2010; Senda et al., 2020; Jiskoot et al., 2023)	95 (Wolpert and Ghahramani, 2000; Roerdink et al., 2009; Bevilacqua et al., 2016; Brodie et al., 2015; Pitale and Bolte, 2018; Baram and Miller, 2007; Tajadura-Jiménez et al., 2015; Gomez-Andres et al., 2020)	39 (Lesur et al., 2025; Stanton and Spence, 2020; Tajadura-Jiménez et al., 2022; Navas-León et al., 2023; Rizzo et al., 2015; Scholz et al., 2015; Potapov et al., 2025; Scholz et al., 2016; Turmo Vidal et al., 2025; Petreca et al., 2023; Ley-Flores et al., 2021a; Botvinick and Cohen, 1998)
Cognitive flexibility	TMT part B [≥41-59^**a**^]	500 (Salbach et al., 2009)	255 (Wolpert and Ghahramani, 2000; Roerdink et al., 2009; Bevilacqua et al., 2016; Brodie et al., 2015; Pitale and Bolte, 2018; Baram and Miller, 2007; Tajadura-Jiménez et al., 2015; Gomez-Andres et al., 2020)	84 (Lesur et al., 2025; Stanton and Spence, 2020; Tajadura-Jiménez et al., 2022; Navas-León et al., 2023; Rizzo et al., 2015; Scholz et al., 2015; Potapov et al., 2025; Scholz et al., 2016; Turmo Vidal et al., 2025; Petreca et al., 2023; Ley-Flores et al., 2021a; Botvinick and Cohen, 1998)
**Functional status**	Scale [Cut-off points to stroke]	**S_01**	**S_02**	**S_03**
Basic ADL	KATZ [≤2/6^**d**^]	3	3	1
Functional independence in ADL	Barthel Index [≥62.90/100^**e**^]	90	100	80
Instrumental ADL	Lawton & Brody [≥8^**f**^]	4	6	7
Fall risk assessment	Risk of falls [>20]	9	5	17
Functional Ability of Lower Limbs	Fugl-Meyer (LL) [<21/34 ^**g**^] for lower mobility funtion	17	19	21
Walking Ability	Modified Walking Ability Categories (Brouwer et al., 2009)	4	4	2
**Emotional well-being**	**Scale** [Presence of symptoms, cut-off points]	**S_01**	**S_02**	**S_03**
Depression symptoms in older adults	Geriatric depression Yesavage scale [≥5/15^**h**^]	4	4	3
Generalized anxiety disorder symptoms	GAD-7 [≥10/21^**i**^]	5	1	2
Self-esteem assessment	Rosenberg Self-esteem Scale [≥15/25^**j**^]	35	29	38

ACE, Addenbrooke's Cognitive Examination; TMT, Trail Making Test; ADL, Activities of Daily Living; GAD, Generalized Anxiety Disorder Scale; Pe, Percentile.

^a^Age-adjusted, NEURONORMA score (Bernat et al., 2015).

^b^Age-adjusted, ACE III, total score (Jiménez-Caballero et al., 2012).

^c^Percentile score in the reference group (Jiskoot et al., 2023).

^d^Cut-off point in stroke (Shelkey and Wallace, 2000).

^e^Cut-off point in stroke (De et al., 2002).

^f^Cut-off point in older adults (Kapoor et al., 2019).

^g^Cut-off point, stroke survivors who score lower than 21 from 34 on the scale should be considered as having lower mobility function using the data-driven approach (Senda et al., 2020).

^h^Cut-off point in Stroke (Park and Kim, 2016).

^i^Cut-off point in Stroke (Tajadura-Jiménez et al., 2012).

^j.^Cut-off point in Stroke (Tajadura-Jiménez et al., 2019).

### 3.2 Outcome measures results

#### 3.2.1 Primary outcomes

##### 3.2.1.1 Gait performance

The percentage change in performance on clinical walking tests from baseline to the end of the “Wind” and “Mechanical” sound intervention periods is presented in Table 4. Figure 3 illustrates individual results on the *10-MWT* and *6-MWT* at the start (T0) and end (T1) of each period. The *10-MWT*, which measures gait speed based on the time taken to walk a 10-meter distance (m/s), showed that exposure to “Wind” sound generally improved walking velocity in all three participants, as reflected by reduced completion times (walking 10 meters). Specifically, S_01 showed a 24.03% improvement in gait speed following the “Wind” sound period, but a 5.75% increase in time after the “Mechanical” sound period. S_02 demonstrated a 20.4% improvement after the “Wind” sound but experienced a 20.75% increase in time following the “Mechanical” sound. Curiously, S_02 was first exposed to the “Mechanical” sound in period B and to then to the “Wind” sound in period C and shows less difference between the two sounds. Finally, S_03 improved gait velocity by 23.9% after the “Wind” sound period and showed a 12.56% increase in time after the “Mechanical” sound exposure period (Figure 3A).

**Table 4:** Percentage of change from Baseline to post-training time in wind and mechanical sound training by subject.

Subject	6-min walking test (m)		10-m walking test (s)	
	Wind sound	Mechanical sound	Wind sound	Mechanical sound
S_01	05.50	08.57	−24.03	05.75
S_02	00.00	−07.81	−20.40	20.75
S_03	92.30	06.79	−23.90	−12.56

The *6-MWT*, which assesses overall functional endurance by measuring the distance walked in 6 min, showed that the “Wind” sound exposure period was effective in improving walking endurance in participants S_01 and S_03. In contrast, the “Mechanical” sound period was generally associated with reduced or minimal improvement in walking endurance. In detail, S_01 improved by 5.50% after the “Wind” sound period but showed a −8.57% decline following the “Mechanical” sound exposure. S_02 exhibited no improvement after the “Wind” condition and experienced a −7.81% decrease after the “Mechanical” condition. S_03 demonstrated a substantial 92.3% improvement in walking endurance after the “Wind” sound period, compared to a more modest 6.79% improvement following the “Mechanical” sound period (Figure 3B).

##### 3.2.1.2 Walking timing symmetry and step frequency

In terms of walking timing symmetry between the affected and non-affected sides, accelerometer data revealed an overall improvement in all three participants from baseline period (period A and A') to the last sound exposure (period B or C), suggesting a carry-over effect of the walking training over the time (Figure 4A). In S_01, significant improvements in timing symmetry were observed from Baseline 1 (A) to Baseline 2 (A') (*z* = 3.68, *p* < 0.001) and the “Mechanical” sound condition (*z* = 4.70, *p* < 0.001). Moreover, timing symmetry improved significantly when comparing the “Wind” condition (period B) to baseline 2 (*z* = 2.43, *p* = 0.015) and to the “Mechanical” sound condition (*z* = 3.54, *p* < 0.001). In S_02, timing symmetry significantly increased from Baseline 1 (A) to the “Mechanical” sound condition (*z* = 4.26, *p* < 0.001), Baseline 2 (A') (*z* = 5.48, *p* < 0.001), and the “Wind” sound condition (*z* = 4.73, *p* < 0.001). S_03 also showed an increase in timing symmetry from Baseline 1 (A) to both Baseline 2 (A') (*z* = 3.99, *p* < 0.001) and the “Mechanical” sound condition (*z* = 3.07, *p* < 0.01). Moreover, symmetry improved when comparing the “Wind” sound condition to Baseline 2 (*z* = 2.80, *p* < 0.01).

**Figure 4:**
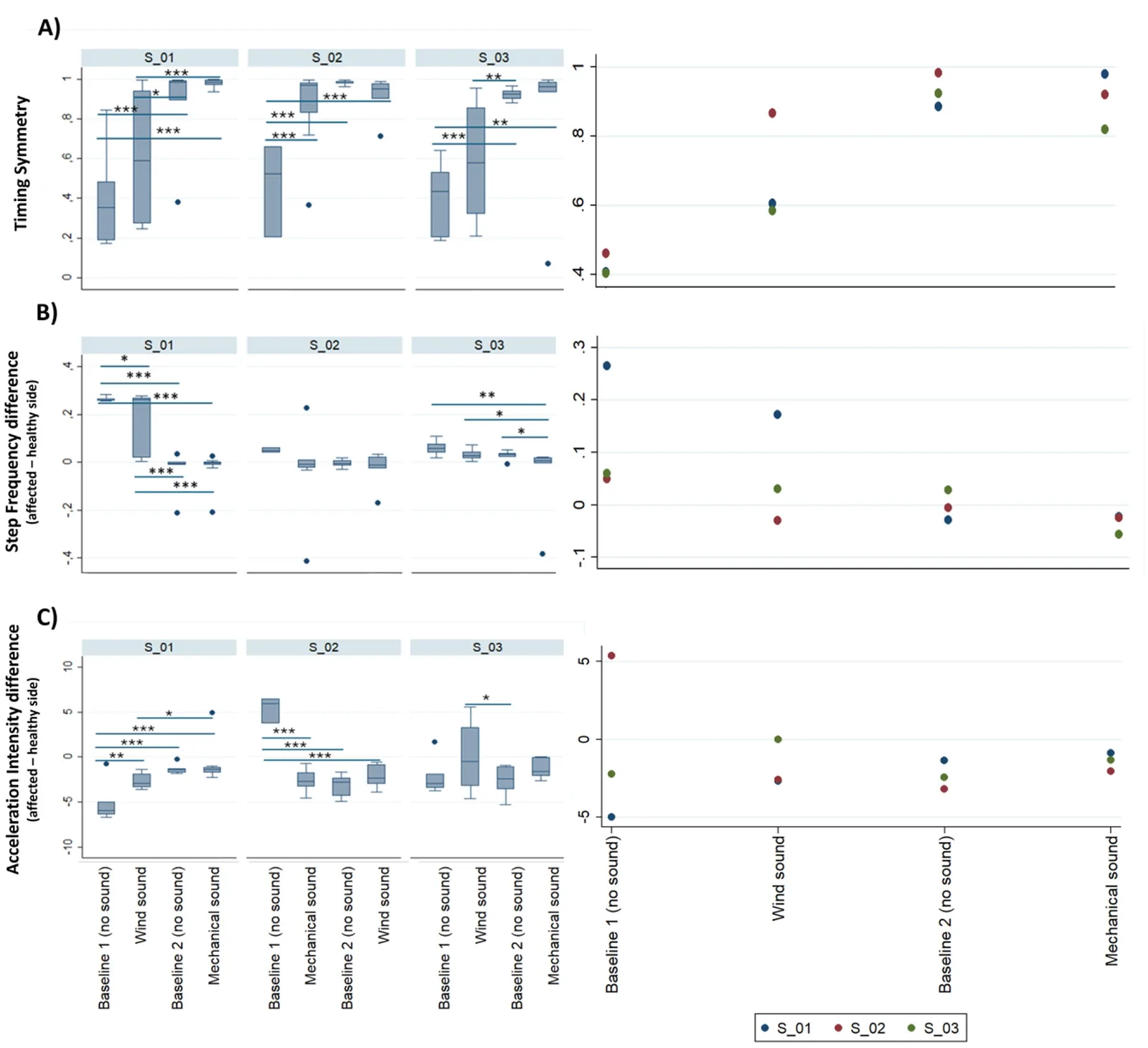
Results of walking parameters from the accelerometers. The right-side panel summarizes the distribution of repeated observations for each walking parameter. The left side panel shows the mean color-coded data points/trajectories by subjects. **(A)** Results from daily walking timing symmetry (msec) measure in each training period. **(B)** Results from daily step frequency measure difference (affected-healthy side) in each training period. **(C)** Results from daily acceleration intensity difference (affected-healthy side) in each training period. Overall, the results show a progressive improvement in all walking parameters from Baseline 1 (no sound) to period C (second sound condition) in all three subjects, showing a carry-over effect from the first sound walking training (period B, first sound condition). In the boxplots, the medians are shown as horizontal lines, and the boxes are the interquartile ranges (IQR). The whiskers are between max (min score, lower quartile−1.5 IQR) to min (max score, upper quartile + 1.5 IQR). If there are values outside the whiskers, these are conventionally called “outliers” and are shown by (°). *p < 0.05, **p < 0.01, ***p < 0.0001.

Regarding step frequency (number of steps) differences between the affected and non-affected sides, results showed a general improvement across all three participants from Baseline 1 (A) to the final period, reflected by a reduction in asymmetry in the number of steps. In S_01, significant improvements were observed from Baseline 1 (A) to the “Wind” sound condition (*z*=1.98, *p*=0.048), Baseline 2 (A') (*z* = −6.10, *p* < 0.001), and the “Mechanical” sound condition (*z* = −6.37, *p* < 0.001). Moreover, step frequency symmetry improved significantly from the “Wind” sound condition compared to both Baseline 2 (A') (*z* = −4.72, *p* < 0.001) and the “Mechanical” sound condition (*z* = −4.98, *p* < 0.001). For S_03, results showed a significant improvement from Baseline 1 (A) to the “Mechanical” sound condition (*z* = −2.76, *p* < 0.01), from the “Wind” to the mechanical sound condition (*z* = −2.21, *p* = 0.027), and from Baseline 2 (A') to the “Mechanical” sound condition (*z* = −2.10, *p* = 0.036) (see Figure 4B).

##### 3.2.1.3 Walking acceleration intensity

Regarding the acceleration intensity difference between the affected and non-affected sides, overall improvements were observed from Baseline 1 (A) to Period C in S_01 and S_02. In S_01, significant improvements were found from Baseline 1 (A) to the “Wind” sound condition (*z* = 2.66, *p* < 0.01), Baseline 2 (A') (*z* = 4.07, *p* < 0.001), and the “Mechanical” sound condition (*z* = 4.92, *p* < 0.001). Moreover, acceleration symmetry also improved between the “Wind” and the “Mechanical” sound condition (*z* = 2.49, *p* = 0.013). For S_02, acceleration symmetry significantly improved from Baseline 1 to the “Mechanical” sound condition (*z* = −10.18, *p* < 0.001), Baseline 2 (*z* = −10.95, *p* < 0.001), and the “Wind” sound condition (*z* = −9.31, *p* < 0.001). However, in S_03 the “Wind” sound condition resulted in significantly greater improvement compared to Baseline 2 (A') (*z* = −2.01, *p* = 0.044) (see Figure 4C).

#### 3.2.2 Secondary outcomes

##### 3.2.2.1 Executive function while walking

Executive function during gait was assessed using the Walking Trail Making Test (WTMT), which evaluates walking performance under attentional and executive load by requiring participants to complete sequential numerical-letters tasks while walking. Overall, results indicated an increase in walking speed (or decrease in walking time) following the Wind sound conditions across all WTMT sub-task. However, S_01 showed the opposite pattern during WRMT-n, with increased walking time and reduced speed. Under the Mechanical sound condition, walking time either increased or remained stable, except for S_01 and S_02, who showed improved walking speed (i.e., reduced time) during WTMT-b (see Table 5, Supplementary Table 1 for further details).

**Table 5:** Scoring performance in walking conditions and dual cognition tasks during gait in the WTMT test.

Conditions	Focused attention				Selective attention				Working memory and cognitive flexibility			
	Wind sound		Mechanical sound		Wind sound		Mechanical sound		Wind sound		Mechanical sound	
Assessment Times	T0	T1	T0	T1	T0	T1	T0	T1	T0	T1	T0	T1
S_01	43	51	22	23	82	65	29	29	182	147	68	63
S_02	14	11	13	16	21	13	17	17	65	24	35	22
S_03	26	17	24	16	36	22	22	24	102	42	38	61

We use raw data to show performance in walking conditions and dual cognition tasks.

##### 3.2.2.2 Sound perception

Results from the sound perception questionnaire indicated that the sound training periods enhanced participants' perception of synchrony between the sound provided by the system and the footsteps and the walking movement compared to the no-sound baseline periods. Specifically, perceived synchrony between sound and footstep movement (Q2) was significantly higher during the “Wind” sound period compared to Baseline 1 (*z* = 2.16, *p* = 0.031) and Baseline 2 (*z* = −4.03, *p* < 0.001). The “Mechanical” sound period also showed higher synchrony than Baseline 2 (*z* = 2.51, *p* = 0.012). In terms of perceived synchrony between sound and ankle movement (Q3), the “Wind” sound condition led to significantly greater perception of synchrony compared to both Baseline 1 (A) (*z* = 4.18, *p* < 0.001) and Baseline 2 (A') (*z* = 2.61, *p* < 0.01). The “Mechanical” sound condition also induced a higher sense of synchrony than Baseline 1 (*z* = 3.67, *p* < 0.001), though not significantly more than Baseline 2. Regarding the perception of walking speed, only the “Wind” sound condition led to a significant increase compared to Baseline 1 (*z* = 2.70, *p* < 0.01). Lastly, in Q6, which assessed the perceived impact of sound on walking rhythm, both sound conditions had a greater impact than the no-sound conditions. The “Wind” sound condition showed a significantly higher impact on rhythm compared to Baseline 1 (*z* = 2.95, *p* < 0.01), while the “Mechanical” sound condition showed a greater impact compared to Baseline 2 (*z* = −2.11, *p* < 0.05). These results are illustrated in Figure 5A.

**Figure 5:**
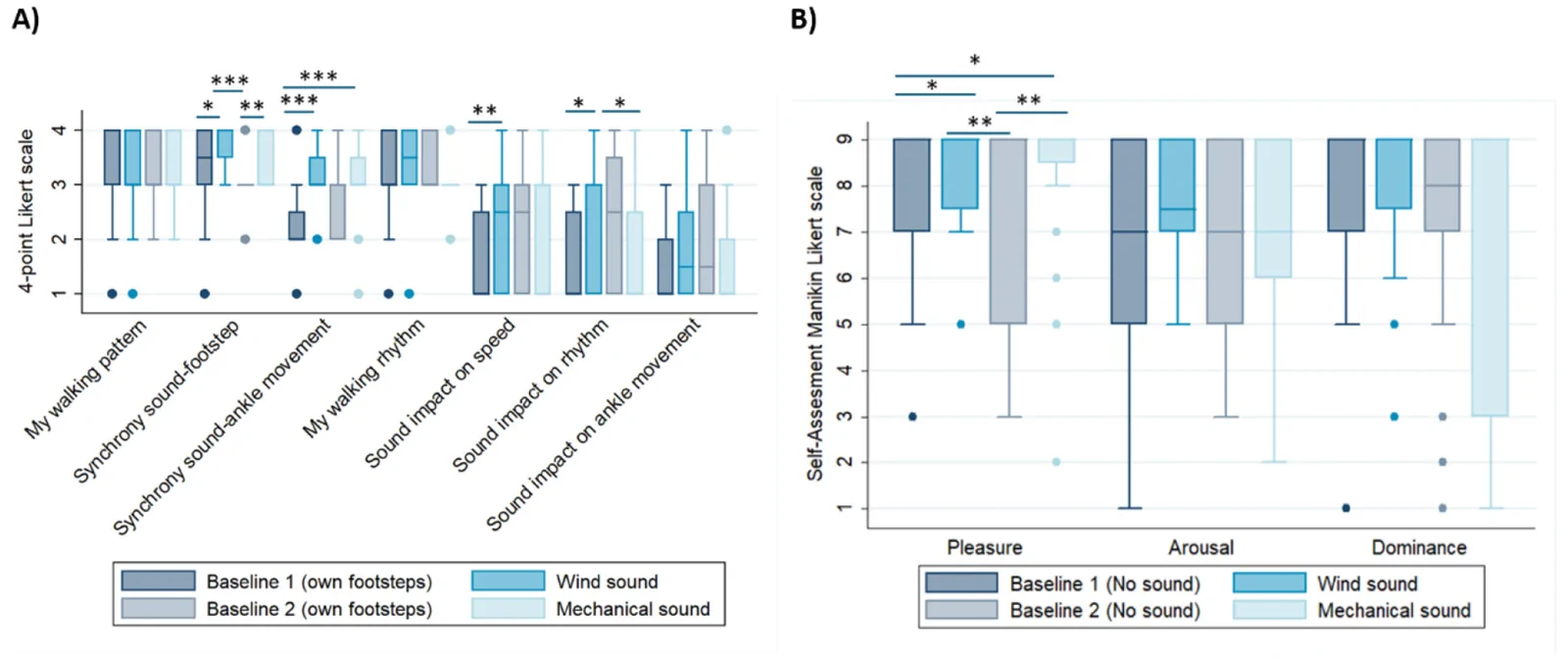
Results of sound perception and the engagement related to the sound training assessment questionnaires. **(A)** Results of the daily sound perception questionnaire after completing each training period. Overall, the results show higher synchrony of the sound input with the footstep and ankle movements in the sound conditions training period (“Wind” and “Mechanical”) compared to the no sound conditions (baseline 1 and 2). Interestingly, the “Wind” sound condition shows a higher impact on walking speed and walking rhythm compared to baseline 1 condition (no sound). **(B)** Results from the Self-Assessment manikin scale related to the pleasure, arousal, and dominance state of the subjects after completing each training period. The results show a high sense of pleasure after the sound conditions training period (“Wind” and “Mechanical”) compared to the no sound conditions [baseline 1 (A) and 2 (A')].

Overall, results from the body and movement feelings questionnaire indicated more positive perceptions related to the body and walking movement following the “Wind” sound period compared to the “Mechanical” sound period (Table 6). Specifically, participants reported higher scores in terms of capability (Q3), flexibility (Q4), and speed (Q5) after the “Wind” sound training, suggesting they felt more capable, flexible, and faster during walking. Additionally, lower scores were reported in Q1, which relates to perceived weight, indicating that participants felt lighter at the end of the “Wind” sound period.

**Table 6:** Mean and SD (standard deviation) of the scoring reported in the body and movement feelings questionnaire.

Sound/Question	Q1. Weight	Q2. Strength	Q3. Capability	Q4. Flexibility	Q5. Speed	Q6. Tiredness	Q7. Control	Q8. Acceleration
Wind	2,33 ± 1,15	5,66 ± 1,15	5,33 ±1,53	6,33 ±1,15	5,66 ±1,15	2,66 ± 2,08	6,33 ± 1,15	4,33 ± 0,58
Mechanical	3,33 ± 2,51	5,33 ± 1,52	3,66 ± 1,53	4,66 ± 1,15	4 ± 0	2 ± 1,73	6 ± 1,73	4 ± 0

##### 3.2.2.3 Emotional state

Results from the Self-Assessment Manikin scale indicated that participants reported significantly higher levels of pleasure during the “Wind” sound training period compared to the no-sound periods: baseline 1 (z = 2.05, *p* = 0.040) and baseline 2 (z = −3.11, *p* = 0.002). Similarly, higher pleasure ratings were reported during the “Mechanical” sound period compared to baseline 1 (z = 2.48, *p* = 0.013) and baseline 2 (z = 3.40, *p* = 0.001) (Figure 5B). While Figure 5 also suggests higher ratings for arousal and dominance during the sound conditions compared to the no-sound periods, these differences were not statistically significant.

Regarding mood, as assessed by the POMS questionnaire, the “Wind” sound training resulted in mood improvements across all dimensions—tension-anxiety, depression-dejection, anger-hostility, vigor-activity, fatigue-inertia, and confusion-bewilderment—with changes ranging from 14 to 17 percent in all participants. In contrast, the “Mechanical” sound condition had no notable effect in S_02 and was associated with a decline in mood state in S_01 and S_03 (see Supplementary Table 2 for further details).

## 4 Discussion

The present multiple case-study, using an ABA'C design, evaluated the potential effect of two types of metaphorical footstep sounds (“Wind,” “Mechanical,”) compared to “Self-produced” natural footstep sounds, in three chronic stroke survivors. The “Wind” and the “Mechanical” sounds were delivered through an interactive walking interface that sonifies movement [SoniBand, (Ley-Flores et al., 2022)], while the self-produced sounds corresponded to participants' natural footsteps. Primary outcomes included gait symmetry, walking velocity, and walking functionality. Secondary outcomes include changes in body perception, emotional state, and cognitive performance following exposure to the different sound conditions (“Wind” and “Mechanical”). Effects of sound conditions were observed across all four domains, even though they should be taken as exploratory due to the small sample size of the study. Specifically, primary outcomes results, showed a greater percentage of change from baseline in walking ability, was observed in all three participants after the “Wind” sound training (Figure 3). In terms of gait symmetry between the affected and non-affected sides, accelerometer data revealed an overall improvement from baseline period to the final sound exposure period (period C), suggesting a carry-over effect of walking training over time. Similarly, steps frequency asymmetry decreased across participants from Baseline 1 (period A) to the final period (period C). Consistent with these results, secondary outcomes results, showed that participants reported feeling more capable, flexible, lighter, and faster after the “Wind” sound condition compared to the “Mechanical” sound condition in the body and movement perception questionnaire. Taken together, these findings suggest that altering footsteps sounds through metaphorical auditory feedback (“Wind” and “Mechanical”) may have a positive impact on walking ability and gait symmetry in chronic stroke survivors. Moreover, the “Wind” sound condition appeared to induce stronger positive bodily feelings than the “Mechanical” sound, particularly in perceived capability, walking velocity, and body lightness.

Previous research has demonstrated that auditory inputs related to the body can modify body and movement perception in both healthy individuals (Tajadura Jimenez et al., 2015; D'Adamo et al., 2024; Tajadura-Jiménez et al., 2017; Lesur et al., 2025; Stanton and Spence, 2020; Tajadura-Jiménez et al., 2022; Navas-León et al., 2023; Rizzo et al., 2015) and clinical populations (Tajadura-Jiménez et al., 2015; Ley-Flores et al., 2021a; Scholz et al., 2015; Potapov et al., 2025; Scholz et al., 2016; Turmo Vidal et al., 2025; Petreca et al., 2023). In healthy participants, studies have shown that sonifying body movements using naturalistic metaphorical sounds (“Wind,” “Water,” “Mechanical” gear, etc.) can influence both body perception and movement quality (Ley-Flores et al., 2022, 2019; Krasny-Pacini and Evans, 2018; Ley-Flores et al., 2021a). The findings of the present study regarding body perception and walking quality after exposure to the “Wind” sound condition align with those of Ley-Flores and co-authors (Krasny-Pacini and Evans, 2018). Similar to their observations, the “Wind” sound employed in the current experiment elicited positive metaphorical associations, enhancing perceptions of body flexibility and lightness, as well as walking speed.

In populations with chronic stroke, previous work by our group investigated walking sonification by manipulating the frequency bands of self-produced natural footstep sounds (Ley-Flores et al., 2021a). That study found significant changes in gait parameters, including reductions in stance and stride time, as well as inversion of asymmetry patterns in heel mean force, when providing amplified natural walking sounds or low frequency bands (83-250 Hz) (Ley-Flores et al., 2021a). In line with these findings, the present study also showed a significant reduction in steps of frequency asymmetry between the affected and non-affected sides after the sonification periods, particularly in two participants (S_01 and S_03). Moreover, similar to Gomez-Andres and colleagues (Ley-Flores et al., 2021a), our study also revealed effects of sound conditions on walking acceleration, as reflected by differences in acceleration intensity between sides. In detail, improvements were observed from Baseline 1 (period A) to Period C in two participants (S_01 and S_03), indicating enhanced acceleration of symmetry. Overall, we can hypothesize that there was an improvement in gait parameters (symmetry and acceleration intensity) across the sonification periods, specifically in S_01 and S_03.

More specifically, improvements in gait performance were observed at the end of the “Wind” sound exposure periods, as assessed by the 10-metersWT and the 6-min WT. Exposure to “Wind” sound generally enhances walking velocity in all three participants, reflected by reduced completion times in the 10-metersWT and by larger percentage changes from baseline. Walking endurance also improved in two participants, as shown by increased walking distance in the 6 min WT at the end of the “Wind” sound exposure periods. However, one participant (S_02) exhibited a decrease in both gait speed and endurance after the “Mechanical” sound exposure. These exploratory findings align with previous investigations showing that movement sonification using “Wind” sounds induces sensations of moving faster and with a lighter body (Ley-Flores et al., 2022; Grau-Sánchez et al., 2017; Krasny-Pacini and Evans, 2018; Ley-Flores et al., 2021a). As described by Turmo Vidal and colleagues (Ley-Flores et al., 2021a), participants may incorporate the metaphorical qualities of sound into their own body (Botvinick and Cohen, 1998). Indeed, these findings are consistent with prior work highlighting the differential impact of the “Wind” and “Mechanical” sound conditions on body movement perception (Ley-Flores et al., 2019; Krasny-Pacini and Evans, 2018; Tsakiris, 2010). The effects of body movement sonification on body perception, walking parameters (symmetry, step frequency, acceleration intensity), and walking ability (walking speed and walking distance) can be understood within the framework of the plasticity and malleability of body experience (Navas-León et al., 2023). Neuroscience research has consistently shown that body experience is continuously updated in response to sensory inputs from the surrounding environment (Tajadura Jimenez et al., 2015; D'Adamo et al., 2024; Matamala-Gomez et al., 2021; Senna et al., 2014; Azanõn et al., 2016). In this context, sound has been proven effective in modulating body representation (Gomez-Andres et al., 2020; Stanton and Spence, 2020; Turchet et al., 2013, 2015), and body movements (Ley-Flores et al., 2022, 2019, 2021a; Linnhoff et al., 2022).

Over the past decade, several studies have applied sound and movement sonification to improve walking in clinical populations (Ley-Flores et al., 2021a; Rodger et al., 2014; Mezzarobba et al., 2018; Wall et al., 2024; Bolt et al., 2021; Dijkerman and Lenggenhager, 2018). A recent review even proposed movement sonification as a promising approach for personalized gait interventions (Maister et al., 2015). These changes in walking patterns induced by auditory input can be interpreted through the Comparator hypothesis or Forward model (Roerdink et al., 2009). According to this, motor performance is influenced when discrepancies arise between predicted and actual sensory feedback. To solve this conflict, the internal model must be readjusted to restore coherence between movement and sensory consequences. This mechanism may explain the walking changes observed in our study, particularly in asymmetry and acceleration of intensity. The system likely responded to discrepancies between predicted and actual sensory input by updating sensory predictions and generating an inverse compensatory adjustment. Such perceived discrepancies, combined with increased uncertainty (i.e., surprise) and the salience of the modified sensory input, may have heightened motor awareness and triggered adaptations in motor planning and execution. These sensorimotor adjustments could account for the improvements observed in walking parameters, including symmetry and step frequency.

We hypothesized that repeated exposure to temporally contingent movement-related auditory feedback could influence gait by reinforcing the association between motor actions and their sensory consequences. The synchronized auditory feedback may provide an additional source of information about the ongoing movement and facilitate sensorimotor integration. Furthermore, the metaphorical characteristics of the “Wind” and “Mechanical” sounds may influence the perceptual and emotional experience of movement. However, because these mechanisms were not directly assessed, they remain hypothetical and should be investigated in future studies.

It is important to mention that in this study other mechanisms can influence the improvements in walking parameters that can operate at a higher cognitive level. It is known that changes in one's body perception (e.g., feeling more flexible or lighter) may broadly impact cognition (van der Hoort et al., 2011; Tajadura-Jiménez et al., 2018; Matamala-Gomez et al., 2019) including spatial aspects such as one's body size (Tajadura Jimenez et al., 2015; Matamala-Gomez et al., 2020; Nava and Tajadura-Jiménez, 2020; Spence, 2011, 2019). For instance, in the Auditory Pinocchio illusion, participants perceive their finger length increasing when an upward pitch glissando sound is heard concurrent to pulling their vertically positioned index finger. As in the well-known Rubber Hand Illusion study (Matamala-Gomez et al., 2021), such illusion emerges when the finger of the participants is hidden from view, while it is vertically extended, and either the experimenter (Keus et al., 2012) or participants themselves (Stanton and Spence, 2020) pull it upwards synchronously to the upward pitch glissando. The sound was displayed by headphones, when a tactile sensor applied to the participants‘ finger was triggered to playback the sound. The paradigm is based on conceptual mappings articulated in the vast literature on cross-modal correspondences (Roel Lesur et al., 2025; Lesur et al., 2025; Schmitz et al., 2018). These mappings refer to the linking of different perceptual features for which the same conceptual label exists. We use spatial terminology to refer to “high”/ low” pitch and “ascending”/ “descending” tones, and people map pitch onto spatial height (95). In the current study, the concepts “Wind” and “Mechanical” may link to features of body movement perception “Flexible” or “Not Flexible”). Similarly, as in the study from Roel Lesur and colleagues [96], in our study different top-down (i.e., attention to sound, sensory imagery) and bottom-up (i.e., different multisensory couplings) cues contributed to the obtained changes on body perception, walking parameters (symmetry, step frequency, acceleration intensity), and walking ability (walking speed and walking distance).

As noted, movement sonification techniques have already been evaluated in clinical populations (Maister et al., 2015), and specifically in stroke survivors (Ley-Flores et al., 2021a; p. 97), typically using experimental group designs that compare two groups at pre- and post- intervention. Building on this evidence, the aim of the present study was to explore the progression of walking recovery and possible changes in body perception through movement sonification, using multiple-case methodology, following single-case study design (Dalrymple-Alford et al., 2010). This methodological approach, frequently applied in clinical psychology, rehabilitation and education, is well suited to generate and test therapeutic mechanisms, as it is particularly sensitive for controlling individual differences and allows each participant to serve as their own control (Dalrymple-Alford et al., 2010). Nevertheless, single and multiple-case designs have limitations, including potential order effects and the difficulty of blinding participants and therapists in behavioral interventions. In the context of stroke rehabilitation, we dealt with irreversibility and carry-over effects, as clear reversals in withdrawal periods are often not observed. However, the absence of reversal in most outcome measures should be interpreted cautiously. Although the maintenance of improvements after withdrawal of the sound conditions may be clinically encouraging and could suggest a persistent training-related effect, the lack of reversal limits the ability to attribute the observed changes specifically to the metaphorical sound intervention. Importantly, significant improvements were also observed between the two baseline periods, during which no sound was presented, suggesting that repeated practice, the structured gait training, or other time-related factors may have contributed to the observed changes. The ankle-worn IMU-based gait metrics in populations with upper motor neuron syndrome, that should be state cautiously, especially the observed changes in acceleration symmetry, which should be interpreted as indicative rather than definitive evidence of motor improvement. Therefore, the present findings cannot disentangle the specific effects of the metaphorical sound stimuli from non-specific effects associated with repeated gait training and the passage of time. Future studies using designs that better control for practice and carry-over effects are needed to determine the specific contribution of metaphorical sound to gait improvement.

## 5 Limitations

The small sample size, commonly used for multiple-case study designs (reference), limits the generalizability of the results, even if it provides relevant information to use new metaphorical sound conditions (“Wind” and “Mechanical”) for walking rehabilitation. The experimental design relies on a crossover rather than a controlled design, which limits the generalizability of the findings to broader walking rehabilitation contexts. Individual variability between participants could strongly influence the observed outcomes due to the small sample size. Moreover, there ia a lack of age-related heterogeneity of the participants. However, the purpose of this exploratory study was not to compare age-defined subgroups or to test differential hypotheses according to biological age. Further adequately powered studies should examine whether age moderates the response to the different acoustic conditions with a larger sample of patients. Another limitation of this study is the relatively low dose of the intervention. The 15 min sonification sessions represented only a small proportion of participants‘ overall daily walking and physical activity. Therefore, although repeated exposure to sonification was associated with changes in some outcomes, maintained changes cannot be attributed exclusively to the intervention, as participants continued their usual activities outside the training sessions. The optimal intensity, frequency, and duration of movement of sonification remain unknown. Future studies should monitor participants' daily activity and systematically investigate dose–response relationships and the persistence of potential effects over longer periods.

## 6 Conclusion

This multiple-case study examined the progression of motor and functional improvements in walking, as well as changes in body perception, in three chronic stroke survivors exposed to metaphorical footsteps sounds (“Wind” and “Mechanical”) compared to “Self-produced” natural footstep sound. Exposure to the “Wind” sound condition led to improvements in gait symmetry and walking velocity. Further, showed enhanced perceptions of body lightness, speed, flexibility, mood improvements and sense of pleasure compared to the “Mechanical” sound and baseline periods. Although the exploratory design limits generalization, the study provides valuable insights into the implementation of movement sonification techniques using metaphorical sounds for gait rehabilitation. However, it is important to emphasize that these findings should be confirmed with a larger sample and/or a control arm in order to validate the observed effects.

## Data Availability

The datasets presented in this study can be found in online repositories. The names of the repository/repositories and accession number(s) can be found in the article/Supplementary material.
