# Supplementary material for: Wind-like sonification of footsteps for walking rehabilitation in chronic stroke survivors: a multiple case study
Source: Front Psychol. 2026 Oct 20;17:1874994. doi: 10.3389/fpsyg.2026.1874994 (PMC13727385; doi:10.3389/fpsyg.2026.1874994)
Supplement: Supplementary file 1 [file Data_Sheet_1.pdf]

Supplementary Material

Table 1. Performance in walking conditions and dual cognition tasks.

	**Focused attention during gait**						**Selective attention during gait**						**Working memory, cognitive flexibility during gait**					
**Conditions**	**WIND**		**Mean (SD)**	**MECHANICAL**		**Mean (SD)**	**WIND**		**Mean (SD)**	**MECHANICAL**		**Mean (SD)**	**WIND**		**Mean (SD)**	**MECHANICAL**		**Mean (SD)**
	WTTMT-n PRE	WTTMT-n POST		WTTMT-n PRE	WTTMT-n POST		WTTMT-a PRE	WTTMT-a POST		WTTMT-a PRE	WTTMT-a POST		WTTMT-b PRE	WTTMT-b POST		WTTMT-b PRE	WTTMT-b POST	
**S_01**	43.01	51.00	47.01 (5.65)	22.00	23.00	22.50 (0.70)	82.00	65.00	73.50 (12.02)	29.00	29.00	29.00 (0.0)	182.00	147.00	164.50 (24.74)	68.00	63.00	65.50 (3.53)
**S_02**	26.00	17.20	21.60 (6.22)	24.00	16.00	20.00 (5.65)	36.02	22.20	29.11 (9.77)	22.00	24.00	23.00 (1.41)	102.00	41.54	71.77 (42.75)	38.00	61.00	49.50 (16.26)
**S_03**	14.00	11.00	12.50 (2.12)	13.00	15.75	14.38 (1.94)	21.00	13.00	17.00 (5.65)	17.00	16.51	17.00 (5.65)	65.00	24.00	44.50 (28.99)	35.00	21.84	28.42 (9.30)

Note: We use data raw to show the performance in walking conditions and dual cognition tasks. SD=Standard deviation

Table 2. Emotional and affective scales

		S_01		S_02		S_03	
		**Wind**	**Mechanical**	**Wind**	**Mechanical**	**Wind**	**Mechanical**
Self-estem	Rosenberg scale	35-31 (0%)	37-33 (-133%)	29-37 (72%)	37-37 (0%)	38-40 (10%)	39-36 (-300%)
Mood state	Profile of Mood States (POMS)	45-76 (14%)	30-20 (-4%)	40-77 (17%)	34-34 (0%)	46-76 (14%)	41-31 (-4%)
Depression symptom	Yesavage scale	4-3 (25%)	2-2 (0%)	4-6 (-50%)	5-2 (60%)	3-0 (100%)	0-1 (0%)
Anxiety symptom	GAD-7 scale	5-3 (40%)	0-1 (0%)	1-1 (0%)	0-1 (0%)	2-0 (100%)	1-0 (100%)

Note: Each cell reports the difference between Evaluation 1 and Evaluation 2, expressed as a percentage of treatment effectiveness. For the Rosenberg Self-Esteem Scale and POMS, we used the following formula to calculate the percentage of treatment effectiveness:
Formula 1: (Post-treatment value – baseline value) / (Maximum possible value – Baseline value) ×100
This formula was applied because higher scores on this scale indicate higher self-esteem.
In contrast, for the Yesavage Depression Scale and the GAD-7, we applied a different formula, as lower scores on these scales indicate fewer symptoms (i.e., improvement):
Formula 2: (Pre-treatment value – post-treatment value) / (Pre-treatment value – Minimum score) ×100

In emotional and psychological measures, changes in self-esteem were observed in S_02 and S_03 during wind training; S_01 and S_03 experienced a decline during mechanical sound therapy. In relation to mood state, the wind sound therapy benefits all patients between 14-17% in contrast to the mechanical sound that does not generate change in S_02, but in S_01 and S_03 generate a decrease of mood state.

In terms of depressive symptoms, S_01 and S_03 reported improvement during wind sound therapy, whereas symptoms worsened during mechanical sound. Conversely, S_02 showed improvement under mechanical sound and worsening during wind sound. In respect to anxiety symptoms decreased in S_01 and S_03 during wind sound therapy, while S_02 maintained a non-anxious status.
